# Ex Vivo Gene and Cell Therapy in Hematopoietic Stem Cells

**DOI:** 10.3390/ijms262311466

**Published:** 2025-11-26

**Authors:** Irina O. Petrova, Svetlana A. Smirnikhina

**Affiliations:** Laboratory of Genome Editing, Research Centre for Medical Genetics, Moskvorechye 1, 115478 Moscow, Russia

**Keywords:** hematoietic stem/progenitor cells, viral vectors, CRISPR/Cas, gene therapy

## Abstract

Ex vivo cell and gene therapy is a prospective approach to treatment of genetic diseases. To date, one of the most prevalent examples of genetically engineered cell therapies is hematopoietic stem/progenitor cells (HSPCs). This mini review is focused on HSPC therapy methods that have been approved for medical use. Most gene therapy methods rely on the lentiviral integration of the gene into the target cell genome, as lentiviruses are extremely effective, particularly in transduction of non-dividing cells. In this constantly evolving field, it is important to find the balance between safety concerns and efficiency. Analyzing cases of several diseases, for which ex vivo gene therapy was developed, we strive to understand which factors are crucial to success and what the potential drawbacks are. Although in general, viral gene integration demonstrates a considerable therapeutic effect, it has oncogenic potential. Development of self-inactivating vectors was a breakthrough in regard to safety, but the possibility of oncogenesis remains, and strict analysis of integration sites is required.

## 1. Introduction

Progress in medical genetics has enabled not only the elucidation of root causes of multiple genetic disorders, but the development of therapies to address these root causes directly. Gene therapy is a method specifically designed to correct the pathogenic genetic variant that leads to a disease, either by directly editing the mutated gene itself or delivering functional variants or other therapeutic molecules that can compensate for the mutation. Alternatively, gene therapy might be used to develop a new functionality in donor cells, particularly immune cells [1]. The most prevalent examples of genetically engineered cell therapies are chimeric antigen receptor T cells (CAR-Ts) and hematopoietic stem/progenitor cells (HSPCs) [2]. Currently existing gene therapy approaches modify CD34+ HSPCs, a heterogeneous population that contains less than 1% stem cells with long-term multi-lineage engraftment potential [3].

In ex vivo gene therapies, the patient’s own cells (autologous) are isolated and modified outside the body to either deliver a functional gene copy or to modify the genome sequence by gene editing. As compared to allogeneic hematopoietic stem cell transplantation, ex vivo gene therapy has the advantages of the absence of a donor requirement and lower toxicity [4].

Another interesting feature of cell genome modification is a possible selective advantage for survival of edited cells. If genome editing restores the function of genes, which is important for cell viability, the edited cell has an advantage over non-edited ones. When such an advantage exists, it reduces the necessary efficiency of modification. One example is gene therapy of X-linked severe combined immunodeficiency, where *IL2RG* gene expression provides progenitors of T cells and natural killer cells with a selective growth advantage [5]. Unfortunately, not all genome edits produce a significant selective advantage. If the edit in question does not improve cell viability, the edited cells do not have an advantage. On the other hand, activation of oncogenes also provides a selective advantage, posing a high risk of tumorigenesis caused by treatment [6,7,8].

Ex vivo gene therapies cause less severe immune responses due to the absence of exposure of the host to the vector [9]. They are also more controllable, which is important for safety reasons. Examples of gene therapies that have currently passed clinical trials and entered the market are presented in Table 1.

## 2. Lentiviruses

As can be seen in Table 1, most existing ex vivo cell therapy methods rely on viral vectors for gene replacement therapy, either by γ-retroviruses or lentiviruses. Lentiviruses and γ-retroviruses contain an RNA genome that is converted to DNA in the transduced cell by a viral reverse transcriptase; after that, viral integrase binds the resulting cDNA to the host DNA and inserts the viral genome into the host genome [10]. In this way, lentiviruses can integrate a large DNA sequence (8–10 kb) into the genome of the host cell [11]. As compared to γ-retroviruses, lentiviruses are able to translocate across the nuclear pore of an intact nuclear membrane and integrate their payload into the genome of non-dividing cells [12]. Because of this, lentiviruses have a preference for actively transcribed genes as integration sites; this is because actively transcribed genes are predominantly located closer to the nuclear pores [10,13]. Cells transduced in their quiescent state retain greater functional potential, and the genetic modification persists to a greater extent.

In multiple clinical trials, the use of γ-retroviral vectors has resulted in insertional mutagenesis, which causes subsequent leukemogenesis and myelodysplastic events [14,15]. A decrease in oncogenic potential by targeting non-dividing cells with lentiviral vectors is possible. Newer lentiviral vectors were designed to reduce the risks of insertional oncogenesis. Recently, however, cases of hematological cancer have been described in patients after lentiviral-transduced CD34+-cell anti-cerebral leukodystrophy therapy due to vector insertions in *MECOM* or *PRDM16* oncogenes [16]. More data needs to be accumulated to assess the comparative risks of retroviral and lentiviral vectors in full.

Self-inactivating (SIN) lentiviral vectors are created using a deletion in the enhancer/promoter of the U3 region of the viral long terminal repeat (LTR) [17]. This deletion prevents the activation of expression in the vicinity of integration sites by viral LTR. Specific promoters are included in the vector to drive the expression of the transgene. SIN lentiviral vectors were found to be more effective in transduction of HSPCs than retroviral vectors [18]. The aim of lentiviral vector design was to improve safety of insertion compared to earlier γ-retroviral vectors.

CD34+ HSPCs are obtained either from bone marrow or by apheresis using flow cytometry or magnetic beads based on surface marker expression. The use of peripheral blood is preferred, as it is a less invasive procedure, which is characterized by a higher stem cell yield and reduced discomfort for donors [19]. The patients receive nonmyeloablative busulfan conditioning after stem cell harvest, which is currently the standard in ex vivo gene therapy [20]. The cells are transduced with lentiviral or retroviral vectors under validated standard operating procedures.

## 3. Genome Editing

Another approach is CRISPR/Cas genome editing. This well-attested and routinely used CRISPR/Cas9 system is a complex consisting of Cas9 nuclease and a single-guide RNA (sgRNA), which matches up with the target sequence [21]. The DNA sequence targeted for editing is termed the protospacer. When the Cas9-sgRNA complex recognizes an NGG protospacer-adjacent motif (PAM) sequence, the spacer (a protospacer-complementary RNA sequence in sgRNA) pairs with the target DNA strand. The Cas9 nuclease creates a blunt-end double-strand break 3 bp upstream of the PAM into the protospacer. The advantage of gene editing is that it preserves the normal copy number and upstream and downstream genomic context. This is important because strict regulation of expression is necessary to maintain the physiological function of genes.

The major drawback for CRISPR/Cas editing is its ability to create only short edits (substitutions, insertions, or deletions) in the strictly determined sequence. Although such editing is highly precise and diminishes the off-target effect, this means that the editing tool should be tailor-made for the particular pathogenic variant, which should be a single small mutation. In the case of a complete loss of a gene copy or a major deletion, this approach cannot be used. There are, however, diseases that have single-point mutations as their only cause, and for these diseases, CRISPR/Cas therapy has great potential. Also, we might expect the development of novel genome-editing-based therapies using CRISPR-directed integrases, which are able to insert up to 36 kb into the host genome [22].

## 4. The Overview of Ex Vivo Gene Therapies

**Figure 1:**
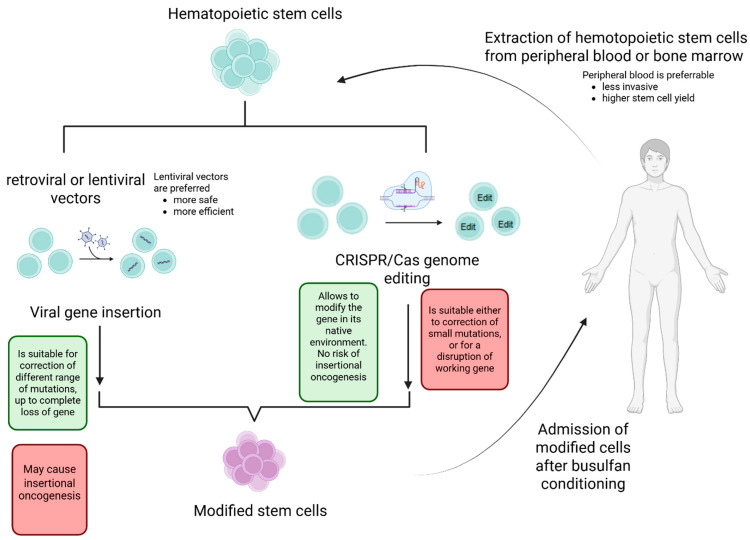
Ex vivo cell and gene therapy using hematopoietic stem cells. The cells are acquired by apheresis or from bone marrow, sorted by flow cytometry or immunobeads, and modified using either viral gene insertion via γ-retroviral or lentiviral vectors or CRISPR/Cas genome editing. The modified cells are administered back to the donor. (Created in Biorender. Irina O. Petrova. (2025) https://BioRender.com).

A general overview of ex vivo HSPC-based gene and cell therapy is presented in Figure 1.

There are currently clinical trials underway for ex vivo lentiviral gene therapy to address the following diseases: chronic granulomatous disease (NCT03645486), hemophilia A and B (NCT03818763, NCT03217032, NCT03961243), multiple variants of severe combined immunodeficiency disorder (NCT03601286, NCT04797260 and others), B-cell lineage malignancies (NCT06116110), Fanconi anemia subtype A (NCT03157804), and many others. Ex vivo CRISPR/Cas gene therapies are being developed for the following diseases: metastatic melanoma (NCT06783270), multiple variants of myeloma (NCT03399448), synovial sarcoma (NCT03399448), and others.

In the following sections, examples of the better-developed gene therapies are described: X-linked immunodeficiency, Wiskott–Aldrich syndrome, adenosine deaminase deficiency, and cerebral adrenoleukodystrophy.

## 5. X-Linked Immunodeficiency

X-linked severe combined immunodeficiency (X-SCID) is a hereditary X-linked disease caused by a deficiency of the common γ (γc) chain of cytokine receptors [23], which is a common component of multiple cytokine receptors. γc is encoded by the *IL2RG* gene. It is involved in multiple cytokine signaling pathways and is crucial for normal development of T cells and natural killer cells. Therefore, γc deficiency causes a major immunity disorder, which is fatal during the first year of life.

X-SCID was one of the first diseases for which gene therapy was developed. Gene replacement therapy was based on the γ-retroviral vector carrying the *IL2RG* gene sequence used for ex vivo transduction of CD34+ hematopoietic stem cells. A defective Moloney murine leukemia virus was used [5,24] to produce the vector, which was then applied for transduction of autologous CD34+ cells. The initial results were promising, as the edited cells were able to overcome the hematopoietic development block and expand relative to their diseased counterparts, producing mature T cells. Development of a functional immune system was reported [7]. Unfortunately, several cases of T cell acute lymphoblastic leukemia were detected later in this clinical trial [6,7,8]. The analysis found insertions in the *LMO2* oncogene locus and in the *CCND2* locus. *LMO2* is a known hotspot for γ-retroviral integration in CD34+ cells [25], and its abnormal expression is strongly associated with T cell leukemia. The fact that viral vectors included intact LTRs, which served as enhancers for *IL2RG* expression, could play some part in the activation of oncogenes. Clonal expansion favored the cell clones carrying the insertion into oncogenic sites.

This regrettable case demonstrated the risks of γ-retroviral gene therapy. After that, the attention of researchers switched to self-inactivating lentiviral vectors, which, notably, do not include intact LTRs. They include physiological gene promoters. As evidence of successful clinical outcomes for clinical trials using SIN lentiviral vectors was obtained [26,27], the development of lentiviral gene therapy for X-SCID was only a matter of time. There were no reports of leukemia cases in clinical trials of SIN lentiviral vectors [28,29,30]. Clinical trials for lentiviral-based gene therapy for X-SCID are currently underway (NCT03601286).

## 6. Wiskott–Aldrich Syndrome

Wiskott–Aldrich syndrome (WAS) is a hereditary X-linked disorder caused by mutations in the *WAS* gene. Its main symptoms are immunodeficiency, eczema, and a reduced ability to form blood clots due to thrombocytopenia. The product of the *WAS* gene is the Wiskott–Aldrich syndrome protein (WASp), which is involved in many functions in hematopoietic stem cells.

Gene replacement therapy for WAS was developed on the basis of a gibbon ape leukemia virus (GALV)-γ-retroviral vector [31]. The early clinical trials demonstrated successful correction of most symptoms of the disease. However, alongside cases of X-SCID, cases of T cell acute lymphoblastic leukemia were discovered later [32]. Later analysis demonstrated integration at the *LMO2*, *MDS1,* and *MN1* loci. Clonal expansion, which was supposed to favor modified cells, promoted the population with activated oncogenic sites.

As in the case of X-SCID, the next step was the use of self-inactivating lentiviral vectors with an endogenous promoter [33]. Clinical trials have been underway since 2013 [34] and have demonstrated clinical efficacy. Importantly, no adverse effects from the use of lentiviral vectors, as well as no cases of leukemia and no clonal dominance, have been detected in these trials. On the other hand, not all symptoms have been corrected with lentiviral therapy, as reconstitution of the platelet count (correction of thrombocytopenia) was not reported [27].

## 7. Adenosine Deaminase Deficiency

Adenosine deaminase (ADA) deficiency is a hereditary disorder inherited in an autosomal recessive manner. It is caused by a mutation in the *ADA* gene. Adenosine deaminase is involved in purine metabolism. Its dysfunction results in the accumulation of toxic metabolites, which disrupts the normal development of the immune system, causing severe combined immunodeficiency (ADA-SCID). Gene therapy based on the γ-retroviral vector derived from the Moloney murine leukemia virus was developed for ADA-SCID [35]. This treatment was clinically efficient, demonstrating long-term gene correction in T lymphocytes, a sustained increase in lymphocyte counts, immune reconstitution, and continued physical growth [36].

Still, a case of T cell leukemia was reported in clinical trials due to insertion in the *LMO2* oncogenic site [37]. This result confirms the risk of oncogenesis due to the insertional activation of oncogenes by γ-retroviral vectors.

The development of SIN lentiviral gene therapy for ADA-SCID is in process. The lentiviral vector used in this therapy contains a short-form elongation factor-1α promoter. The high overall survival and lack of adverse events at 24 and 36 months are promising and compare favorably with the results of HSCT [38]. Still, further research is needed to confirm safety and efficacy of lentiviral treatment.

## 8. Cerebral Adrenoleukodystrophy

Cerebral adrenoleukodystrophy (CALD) is a hereditary X-linked metabolic genetic disease. It is caused by mutations in the *ABCD1* gene, which encodes the adrenoleukodystrophy protein (ALDP), an ATP-dependent transporter, which is involved in peroxisomal import of fatty acids and/or fatty acyl-CoAs. Its dysfunction leads to the accumulation of very long-chain fatty acids, which negatively affect the adrenal cortex, brain, and spinal cord white matter. Therefore, CALD is characterized by symptoms of various severity, ranging from adrenal dysfunction to degeneration to a vegetative state. Elivaldogene autotemcel (SKYSONA™; eli-cel; Lenti-D™) is a gene therapy that has been developed by bluebird bio for the treatment of CALD [bluebird bio. From 10-K. 2021. https://investor.bluebirdbio.com/static-files/a0cfdff2-2784-4e01-9237-8be586e10bce. Accessed on 7 July 2021]. It is based on the Lenti-D lentiviral vector encoding the human *ABCD1* cDNA under the control of the Moloney leukemia virus long terminal repeat (MNDU3) promoter-enhancer that includes the U3 segment of the myeloproliferative sarcoma virus long terminal repeat with the negative control region deleted and the DL587 endogenous retrovirus primer binding site substituted. Lenti-D is a SIN lentiviral vector pseudotyped with glycoprotein G of the vesicular stomatitis virus.

Clinical trials of elivaldogene autotemcel were successful, and adverse effects were low [39].

Elivaldogene autotemcel received its first approval on 16 July 2021 for the treatment of CALD in patients < 18 years of age, with an *ABCD1* genetic mutation, and for whom a human leukocyte antigen-matched sibling hematopoietic stem cell donor is not available. Bluebird bio received the European Commission approval for SKYSONA™ (elivaldogene autotemcel, Lenti-D™) gene therapy for patients less than 18 years of age with early cerebral adrenoleukodystrophy (CALD) without a matched sibling donor, but on 18 November 2021, the European Commission withdrew the marketing authorization for elivaldogene autotemcel in the European Union.

The noted complication in elivaldogene autotemcel is the occurrence of autologous anti-ALDP antibodies, which leads to the loss of modified cells [40] in patients with full gene loss.

Cases of hematological cancer were reported in patients who received elivaldogene autotemcel treatment [16]. The analysis identified insertions in multiple loci, including ECOM-EVI1 (MDS and EVI1 complex protein EVI1 [ecotropic virus integration site 1]) or PRDM16 (positive regulatory domain zinc finger protein 16). Clonal expansion favored such variants. This finding confirms that extensive research of vector integration profile and careful choice of regulatory elements is required for safety of gene replacement therapy.

## 9. β-Thalassemia and Sickle Cell Disease

The human β-globin locus encodes five globin genes: the embryonic ε gene (*HBE1*), the 2 fetal Gγ and Aγ genes (*HBG2*, *HBG1*), and the adult δ and β genes (*HBD*, *HBB*), expressed in erythrocytes in the bone marrow and blood after birth. Each gene is under the control of a specific promoter [41]. A regulatory region called the 5′ locus control region (LCR) is found 6–40 kb upstream of *HBE1.* It consists of 5 sites: HS1, HS2, HS3, HS4, and HS5.

The prevalent form of hemoglobin during fetal development is called fetal hemoglobin (HbF) and consists of two α-globin and two γ-globin chains. After birth, it is replaced with adult hemoglobin (HbA), consisting of two α-globin and two β-globin chains.

β-Thalassemia is caused by mutations in the β-globin gene (*HBB*) that either reduce (β+) or fully suppress (β0) production of functional β-globin [42]. An excess of unpaired α-globin impedes development and survival of erythrocytes, leading to ineffective erythropoiesis, hemolysis, chronic anemia, and compromised quality of life [43].

Sickle cell disease (SCD) is caused by a single A→T (E6V) point mutation in the *HBB* gene, which leads to the production of sickle hemoglobin (HbS). Sickle hemoglobin, unlike normal hemoglobin, is prone to aggregation, which leads to impaired function of erythrocytes [44]. Less severe symptoms of β-thalassemia and sickle cell disease were observed in patients who did not undergo this transition and maintained a high level of HbF in the adult state [45], because γ-globin demonstrates anti-sickling activity.

Multiple lentiviral vectors carrying the *HBB* gene sequence were developed for gene therapy of β-thalassemia and sickle cell disease. The first vector, TNS9, carried a β-globin cassette including introns with an intron 2 internal deletion under control of an endogenous β-globin promoter and a 3.2 kb locus control region (LCR) element (HS2, HS3, and HS4) [46]. This vector was not designed for treatment of sickle cell disease. To solve this problem, other vectors were developed, which carried the *HBG1* or *HBG2* gene under control of the minimal β-globin promoter and selected parts of a 2.0 kb LCR with HS2, HS3, and HS4 regulatory elements [47]. These vectors were designed for expression of γ-globin and subsequent formation of HbF, which is resistant to sickling.

An alternative approach is to introduce mutations into the *HBB* sequence that prevent sickling. The first mutations to be employed include a single T87Q mutation in exon 2 [48]. Later, two more amino acid substitutions (E22A and G16D in exon 1) were introduced to create β^AS3^-globin [49].

The next development was the LentiGlobin HPV569 lentiviral vector, an SIN lentiviral vector carrying two copies of the 250 bp core sequence of the chicken 1.2 kb hypersensitive site-4 (cHS4) chromatin insulator in the 3′ LTR to insulate a promoter from the possible effect of an upstream enhancer and to reduce position effects. However, cHS4 elements were proven not to be as beneficial as previously believed [50]. In the next generation of LentiGlobin vectors, cHS4 elements were removed to enhance transduction capabilities. Also, the wild-type HIV1 U3 region was replaced with a CMV promoter in the 5′ LTR. The resulting globin vector construct is called the LentiGlobin BB305 lentiviral vector.

The LentiGlobin BB305 vector carrying the β^A-T87Q^-globin gene sequence under control of the endogenous β promoter with HS2, HS3, and HS4 regulatory elements was used for development of betibeglogene autotemcel (beti-cel; Bluebird Bio) [51]. This therapy demonstrated high clinical efficacy and allowed most patients with transfusion-dependent β-thalassemia and a non-β0/β0 genotype to reach transfusion independence [52]. No cases of insertional oncogenesis and malignancies were reported [53].

The LentiGlobin BB305 vector encoding a human β^A-T87Q^ was found to be efficient in the case of SCD [54]. Gene therapy using this vector was registered as lovotibeglogene autotemcel (Bluebird Bio). One-time lovotibeglogene autotemcel treatment resulted in sustained production of β^A-T87Q^ in most erythrocytes, reduced hemolysis, and complete resolution of severe vaso-occlusive events. (Funded by Bluebird Bio; HGB-206 ClinicalTrials.gov number, NCT02140554). Two cases of T cell leukemia were reported after lovotibeglogene autotemcel treatment, but analysis of insertion sites showed that insertional oncogenesis is unlikely [55].

An alternative gene therapy for SCD was developed with the use of γ-globin-carrying vectors. The GGHI-mB-3D γ-globin lentiviral vector uses HbF induction via the incorporation of elements associated with hereditary persistence of fetal hemoglobin. It does not contain LCR elements but comprises novel regulatory elements from the γ- and α-globin loci (HPHF-3D, HPHF-2, HS40 enhancers) [56,57].

A completely different approach was demonstrated by exagamglogene autotemcel (exa-cel). It is a non-viral therapy based on ex vivo CRISPR/Cas editing aimed to reactivate γ-globin expression. CRISPR/Cas ribonucleoprotein carrying sgRNA for erythroid-specific enhancer region of the *BCL11A* gene is delivered by electroporation into autologous CD34+ HSPCs [57]. *BCL11A* is a transcription factor that represses the expression of γ-globin after birth [58]. Electroporation is a highly effective delivery method, but it is characterized by considerable cytotoxicity due to transient membrane disruption and potential non-reversible membrane permeabilization. It is employed in exa-cel because this method relies not on insertion of a functional gene copy into the host genome, which is the mechanism of action of lentiviral vectors, but on the modification of the existing gene. Lipid nanoparticles are used for RNA or RNP delivery, demonstrating less toxicity, so we might expect development of novel gene editing cell therapy methods based on lipid nanoparticles [59,60].

In the case of exa-cel, genome editing is employed to disrupt the functional gene, but not to perform a precise homology-driven edit. In cases where diseases are caused by complete loss of genes, a DNA template would be necessary to restore the correct gene sequence by homology-driven repair. Therefore, the delivery of dsDNA template along with sgRNA and Cas (either as a protein or as an mRNA) would be necessary, adding further methodical complications.

Treatment with exa-cel was clinically effective. Exa-cel has, therefore, been licensed for the treatment of SCD or transfusion-dependent β-thalassemia in patients 12 years of age and older.

## 10. Metachromatic Leukodystrophy

Metachromatic leukodystrophy (MLD) is a rare genetic disease inherited in an autosomal recessive manner. It is caused by mutations in the *ARSA* gene that encodes arylsulfatase A (ARSA), which is an enzyme that breaks down sulfatides. Its dysfunction causes the accumulation of sulfatides, which negatively affects the nervous system [26,61]. A gene therapy based on lentiviral vectors carrying *ARSA* cDNA under the control of the human phosphoglycerate kinase gene promoter was developed and registered under the name atidarsagene autotemcel (arsa-cel). The first clinical trials were successful [26]. Unfortunately, the treatment was effective only in early-onset cases of the disease. Clearly symptomatic patients suffer from consequences of sulfatide accumulation, which cannot be alleviated by gene replacement therapy [39].

## 11. Conclusions

Integration of the lentiviral gene remains the most successful variant of ex vivo gene therapy. A single example of FDA-approved CRISPR/Cas-based therapy (exa-cel), interestingly, does not rely on the reconstruction of normal gene variants, but on the disruption of a silencing factor to promote the expression of an alternative protein. In the future, development of precise genome editing might lead to the development of new kinds of gene therapy. For now, lentiviral gene integration has the benefits of wider applicability because it is not tied to a particular pathogenic variant. On the other hand, physiologically important genes are under tight regulation, as can be seen in the case of β-globin; in addition, understanding of the genomic context is required to ensure their proper expression after genome integration. The disruption of the genome in the integration site might have dire consequences, as was proven by leukemia cases in the trials with retroviral vectors. The major improvement was creation of self-inactivating viral vectors that drastically reduced the oncogenic effect of retroviral therapy. Nevertheless, lentiviral-based cell therapies were developed only recently and require close monitoring. The recent reports of leukemia cases in eli-cel therapy raise concerns and demand prioritizing safety in design of new viral vectors.

A further avenue of development could be the use of CRISPR/Cas-based genome editing. This approach minimizes the risk of insertional oncogenesis and allows function of the gene to be restored in its functional environment and the physiological regulation of expression to be maintained. Currently, only one gene therapy has received approval and entered the market, but there are more clinical trials underway. The downside is that CRISPR/Cas is able to introduce only minor edits into the gene sequence, so the editor has to be finely tuned for each possible pathogenic variant. Opportunities for improvement lie in the use of more advanced genome editors, such as a prime editor, and in the use of novel methods of delivery, including lipid nanoparticles.

Cell therapy has enormous labor and material costs. The manufacturing process of gene and cell therapy is complex and expensive, limiting its widespread use. Still, its potential for the treatment of monogenic diseases is far from exhausted, with ongoing development continually creating new opportunities.

## Figures and Tables

**Table 1:** Ex vivo cell therapy methods, which have currently entered the market.

Commercial Name	Name	Target Cells	Gene Delivery Method	Disease	Year of Approvement	Approving Agency
Strimvelis	Autologous CD34+ enriched cell fraction that contains CD34+ cells transduced with a retroviral vector that encodes for the human ADA cDNA sequence	Genetically modified autologous CD34+ hematopoietic stem cells	replication-incompetent retroviral vector	Adenosine deaminase deficiency	2018	EMA (https://www.ema.europa.eu/en/medicines/human/EPAR/strimvelis (accessed on 23 November 2025))
Skysona	Elivaldogene autotemcel	Genetically modified autologous CD34+ hematopoietic stem cells	lentiviral vector	Adrenoleukodystrophy	2022	FDA (https://www.fda.gov/vaccines-blood-biologics/skysona (accessed on 23 November 2025))
Zynteglo	Betibeglogene autotemcel	Genetically modified autologous CD34+ hematopoietic stem cells	lentiviral vector	Beta-thalassemia	2022	FDA (https://www.fda.gov/vaccines-blood-biologics/zynteglo (accessed on 23 November 2025))
Lyfgenia	Lovotibeglogene autotemcel	Genetically modified autologous CD34+ hematopoietic stem cells	lentiviral vector	Sickle cell disease	2024	FDA (https://www.fda.gov/vaccines-blood-biologics/lyfgenia (accessed on 23 November 2025))
Casgevy	Exagamglogene autotemcel	Genetically modified autologous CD34+ hematopoietic stem cells	CRISPR/Cas	Beta-thalassemia, sickle cell disease	2024	FDA (https://www.fda.gov/vaccines-blood-biologics/casgevy (accessed on 23 November 2025))
Libmeldy, Lenmeldy	Atidarsagene autotemcel	Genetically modified autologous CD34+ hematopoietic stem cells	lentiviral vector	Metachromatic leukodystrophy	2024	FDA (https://www.fda.gov/news-events/press-announcements/fda-approves-first-gene-therapy-children-metachromatic-leukodystrophy (accessed on 23 November 2025); https://www.fda.gov/vaccines-blood-biologics/cellular-gene-therapy-products/lenmeldy (accessed on 23 November 2025))

## Data Availability

No new data were created or analyzed in this study. Data sharing is not applicable to this article.
